# Conformational Dynamics of *Escherichia coli* Flavodoxins in Apo- and Holo-States by Solution NMR Spectroscopy

**DOI:** 10.1371/journal.pone.0103936

**Published:** 2014-08-05

**Authors:** Qian Ye, Yunfei Hu, Changwen Jin

**Affiliations:** 1 Beijing Nuclear Magnetic Resonance Center, Peking University, Beijing, China; 2 College of Life Sciences, Peking University, Beijing, China; 3 College of Chemistry and Molecular Engineering, Peking University, Beijing, China; 4 Beijing National Laboratory for Molecular Sciences, Peking University, Beijing, China; University of Pittsburgh School of Medicine, United States of America

## Abstract

Flavodoxins are a family of small FMN-binding proteins that commonly exist in prokaryotes. They utilize a non-covalently bound FMN molecule to act as the redox center during the electron transfer processes in various important biological pathways. Although extensive investigations were performed, detailed molecular mechanisms of cofactor binding and electron transfer remain elusive. Herein we report the solution NMR studies on *Escherichia coli* flavodoxins FldA and YqcA, belonging to the long-chain and short-chain flavodoxin subfamilies respectively. Our structural studies demonstrate that both proteins show the typical flavodoxin fold, with extensive conformational exchanges observed near the FMN binding pocket in their apo-forms. Cofactor binding significantly stabilizes both proteins as revealed by the extension of secondary structures in the holo-forms, and the overall rigidity shown by the backbone dynamics data. However, the 50 s loops of both proteins in the holo-form still show conformational exchanges on the µs-ms timescales, which appears to be a common feature in the flavodoxin family, and might play an important role in structural fine-tuning during the electron transfer reactions.

## Introduction

Flavodoxins are a family of small proteins containing a non-covalently bound flavin mononucleotide (FMN) molecule, which is able to switch between three redox states (the fully-oxidized, semiquinone, and hydroquinone states) to function as the redox center in electron transfer reactions [1]. Flavodoxins are widely distributed in prokaryotes and participate in various biological processes, including photosynthesis, methionine synthesis, biotin synthesis, anti-oxidation and enzyme activations [1]–[9]. In most non-photosynthetic reactions, electrons flow from NADPH to flavodoxin reductase and then to flavodoxin, which subsequently transfers the electrons to downstream targets [10]. In eukaryotes, flavodoxins-like domains are present in many multi-domain proteins, and play similar roles in the electron transfer pathways.

Based on protein sequences and three-dimensional structures, flavodoxins can be further classified into two subfamilies, namely the long-chain and short-chain subfamilies. The two subfamilies differ in the presence or absence of a 20-residue insertion, which is not involved in cofactor binding but may play a role in protein-protein interactions [11]–[13]. Although biochemical and structural studies have been extensively carried out for flavodoxins or flavodoxin-like domains, the molecular mechanisms underlying the cofactor binding and electron transfer processes remain elusive.

The *Escherichia coli* genome harbors several genes encoding proteins assigned to the flavodoxin family, whereas experimental evidence suggested that they diverge in biological functions [12], [13]. The *fldA* gene encodes a long-chain flavodoxin FldA, which is essential for bacterial survival and is involved in many biological pathways, such as the activations of pyruvate-formate lyase, ribonucleotide reductase and biotin synthase [4], [5], [14], [15]. The *mioC* gene encodes a short-chain flavodoxin MioC that was identified to be essential for biotin synthesis, but its role in this process is different and irredundant from that played by the FldA protein [16]. Up to date, biochemical and structural studies have been performed on these two proteins to address the molecular mechanism underlying their cofactor binding and electron transfer processes [4], [5], [14]–[18]. The structures of both apo- and holo-forms of MioC were solved and the backbone dynamics were investigated by solution NMR spectroscopy [17]. The structure of *E. coli* FldA in its holo-form has been solved by X-ray crystallography and also subjected to hydrogen-deuterium exchange studies by NMR [15], [18], whereas the apo-form was unable to get crystalized and remain less well characterized. The *E. coli yqcA* gene encodes another flavodoxin of the short-chain subfamily. However, no experimental investigations have been reported thus far and the exact role of the YqcA protein remains to be determined.

In an effort to systematically study the structure-function relationship of the flavodoxin family in *E. coli*, we determined the solution structures of both apo- and holo-YqcA, as well as holo-FldA by high-resolution nuclear magnetic resonance (NMR) spectroscopy. In addition, we investigated the dynamic properties of YqcA and FldA in both forms. These results, together with our previously reported structural and dynamic studies of MioC, provide new insights in understanding the molecular mechanisms of cofactor binding and electron transfer by flavodoxins.

## Materials and Methods

### Protein expression and purification

The *E. coli yqcA* and *fldA* genes were cloned into the pET 21a (+) (Novagen) vector and expressed in *E. coli* BL21 (DE3) strain (Invitrogen). The cells were grown in 1 L Luria–Bertani (LB) broth medium containing 50 mg/mL of ampicillin at 35°C. When the OD_600_ reached 0.8, the cells were centrifuged at 4°C and resuspended in 250 ml of M9 minimal medium with ampicillin and ^15^NH_4_Cl in the presence or absence of ^13^C_6_-glucose for preparations of ^13^C/^15^N-labeled or ^15^N-labeled samples, respectively [19]. After shaking at 35°C for an hour, isopropyl-β-D-thiogalactoside (IPTG) was added to a final concentration of 0.4 mM to induce protein expression. The cells were harvested 8 hr later and the protein was purified by anion-exchange chromatography (Mono Q) followed by gel filtration (Superdex-75) using an ÄKTA FPLC system (GE Healthcare). The purity was identified to be greater than 95% by SDS-PAGE.

### Sample preparation

The YqcA and FldA samples in the apo-form were prepared by precipitation using 5% trichloroacetic acid to remove the FMN molecule, followed by protein refolding in a buffer containing 30 mM sodium phosphate (pH 7.0), 30 mM NaCl, and 20 mM dithiothreitol (DTT) [20]. The holo-form were prepared by refolding the protein in a buffer containing 30 mM sodium phosphate (pH 7.0), 30 mM NaCl, and an excess of FMN (50 mM). ^2^H_2_O (5%) was added into the NMR samples, and 2,2-dimethyl-2-silapentanesulfonic acid (DSS) was added as the internal chemical shift reference.

### NMR spectroscopy

The NMR spectra were acquired at 25°C on Bruker Avance 600 MHz and 800 MHz spectrometers, both equipped with four RF channels and a triple-resonance cryo-probe with pulsed field gradients. The two-dimensional (2D) ^15^N- and ^13^C-edited heteronuclear single quantum coherence (HSQC) spectra, together with the three-dimensional (3D) HNCA, HNCACB, CBCA(CO)NH, HNCO, HN(CA)CO, HBHA(CO)NH, (H)CCH-COSY, and (H)CCH-TOCSY experiments were collected for backbone and side chain assignments [21]–[24]. The 3D ^15^N- and ^13^C-edited NOESY-HSQC spectra (mixing times 100 ms) were performed to confirm the chemical shift assignments and obtain distance restraints for structure calculations. The ^1^H chemical shifts were referenced to internal DSS, and ^13^C and ^15^N chemical shifts were referenced indirectly [25]. All spectra were processed using the software package NMRPipe [26] and analyzed by the program NMRView [27].

### Titration experiments

The FMN titration experiments were performed and monitored by a series of 2D ^15^N-edited HSQC experiments. The samples of apo-YqcA and apo-FldA were first exchanged using the NMR buffer without DTT under anaerobic conditions to remove DTT. The HSQC spectra of the apo-proteins in the NMR buffer with or without DTT were identical. During the titration, the molar ratio of FMN:protein was gradually increased from 0.1∶1 to 2∶1. The spectra were collected on a Bruker Avance 800 MHz NMR spectrometer at 25°C.

### Structure calculations

The structure calculations were performed using the program CYANA [28], [29] and refined by AMBER [30]. Distance restraints were derived from inter-proton nuclear Overhauser effect (NOE). Dihedral angles (φ and ψ) were predicted from chemical shifts using TALOS [31]. The initial structures were generated using the CANDID module of CYANA [29], and 20 structures with the lowest energies were selected as models for the program SANE to extend the NOE assignments [32]. 200 structures were calculated by CYANA, and the 100 lowest-energy structures were further refined by AMBER [30]. Finally, 20 lowest-energy conformers were selected as the representative structures. In the structure calculation of holo-YqcA and holo-FldA, in addition to the intermolecular NOEs identified between the protein and the FMN molecule, distance restraints were added between the phosphate group of the FMN molecule and residues in the phosphate-binding loop (P-loop) based on their significant chemical shift perturbations as previously reported [33], [34]. The final structures were analyzed using the program packages MOLMOL [35] and PROCHECK_NMR [36].

### Relaxation measurements

The backbone ^15^N relaxation parameters, including the longitudinal relaxation rates (*R_1_*), transverse relaxation rates (*R_2_*), and steady-state heteronuclear {^1^H}-^15^N NOE values of the YqcA and FldA in both apo- and holo-forms were measured on a Bruker Avance 800 MHz NMR spectrometer at 25°C [37]. The delays used for the *R_1_* experiments were 10 (×3), 100, 300, 450, 600, 800, 1000, 1300, 1600, 2400, 3200 and 4000 ms for both YqcA and 10 (×2), 100, 300, 700, 1200, 1800, 2500, 3200 and 3990 ms for FldA. The delays used for the *R_2_* experiments were 7.4 (×2), 14.8, 22.3, 37.1, 29.7, 52.0, 74.2, 89.1, 111.4, 148.5, 185.6 and 222.7 ms for YqcA, and 6 (×2), 10, 18, 34, 54, 74, 98, 122 and 162 ms ms for FldA. The relaxation rate constants were obtained by fitting the peak intensities to a single exponential function [38]. The {^1^H}-^15^N NOE experiments were performed in the presence and absence of a 3-s proton presaturation period prior to the ^15^N excitation pulse and using recycle delays of 2 and 5 s, respectively.

## Results

### NMR characterizations of structure and activity of *E. coli* YqcA

The YqcA protein expressed and directly purified from *E. coli* showed an elution with yellowish color, indicating the presence of the FMN cofactor. However, the 2D ^15^N-edited HSQC spectrum showed two sets of peaks, suggesting multiple conformations (Figure S1). We subsequently used trichloroacetic acid precipitation and refolding procedures to remove the FMN cofactor as previously described to obtain the apo-YqcA sample [20]. The 2D ^15^N-edited HSQC spectrum of the apo-YqcA showed a single set of peaks, indicating a unique conformation.

To confirm YqcA is an FMN-binding protein, titration experiments were performed. Upon addition of the FMN molecule, the set of cross-peaks corresponding to the apo-form gradually decreases in intensities and finally disappear, whereas a new set of cross-peaks corresponding to the holo-form appears. We compared the HSQC spectrum of the directly purified YqcA sample with those of the apo- and holo-forms, and confirmed that the directly purified protein contains both forms of YqcA that are in slow exchange with each other. These results demonstrate that YqcA binds FMN with high affinity which is similar to other flavodoxins [15], [17].

Chemical shifts assignments of apo- and holo-YqcA were performed and reported elsewhere [39]. Briefly, the NMR signals for 23 backbone amides were missing for the apo-form, whereas nearly all backbone amide signals showed up for the holo-form. The solution structures of both the apo- and holo- YqcA were subsequently determined using NOE-derived distance restraints in combination with dihedral angle restraints. The coordinates of both apo- and holo-forms of YqcA are deposited in the Protein Data Bank (PDB) under the accession numbers 2M6R and 2M6S, the chemical shift assignments have been deposited in BioMagResBank (BMRB, http://www.bmrb.wisc.edu/) under the accession numbers 19151 and 19152 [39], and the structural statistics are summarized in Table S1.

**Figure 1:**
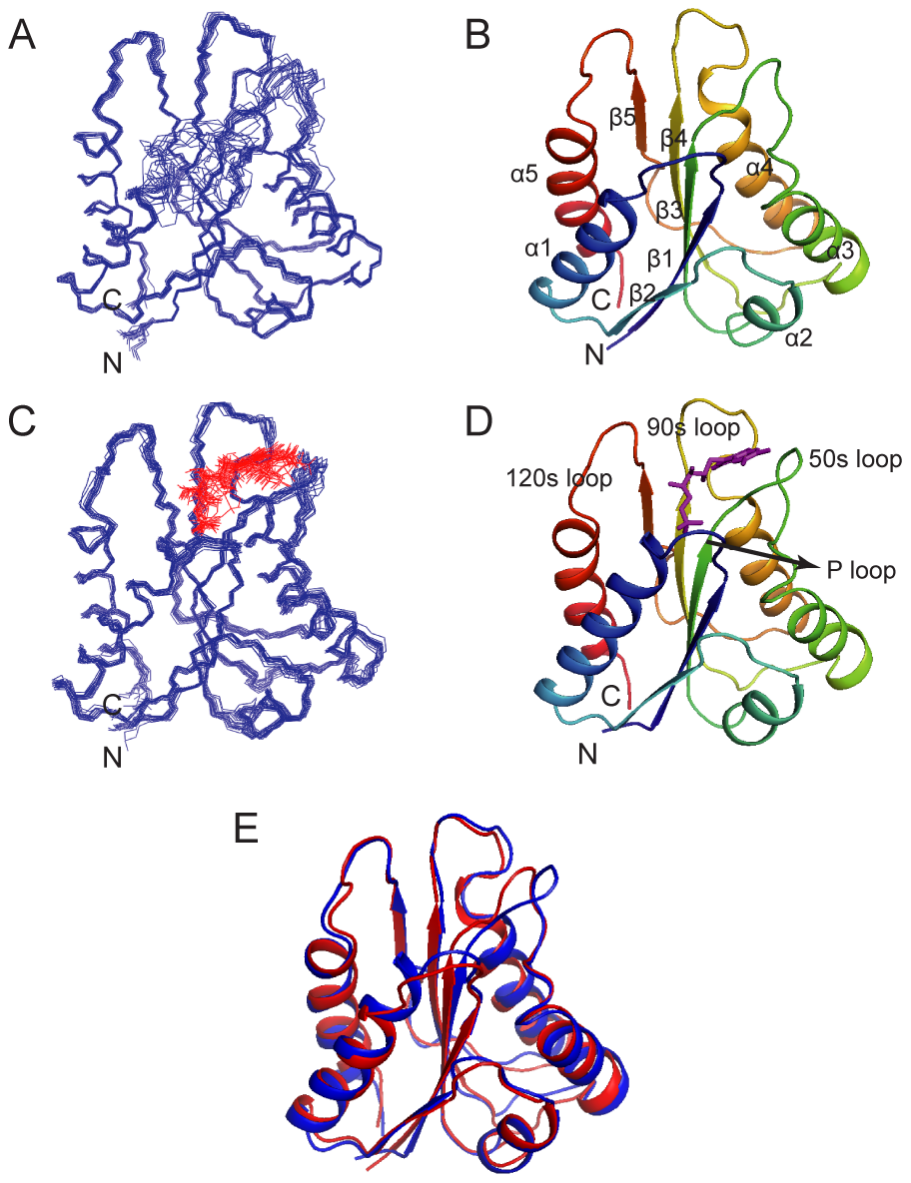
Solution structures of the apo- and holo-forms of *E. coli* YqcA. (A, C) Superimpositions of the 20 representative solution structures of YqcA in the apo- (A) and holo-forms (C). The FMN molecule is shown in red. (B, D) Ribbon diagram representations of YqcA in the apo- (B) and holo-forms (D). The secondary structural elements and the loops around the FMN-binding site are labeled in B and D, respectively. (E) An overlay of the ribbon diagram of apo- (red) and holo-YqcA (blue). The FMN molecule is not shown.

As shown in Figure 1A-D, the YqcA protein shows a typical flavodoxin fold consisting an α/β sandwich with a central five-strand parallel β-sheet (β1: Glu3-Gly9, β2: Lys32-Glu37, β3: Tyr51-Thr56, β4: Arg86-Gly93, β5: Met122-Asp126) flanked by five α-helices (α1: Asn14-Gln29, α2: Leu41-Tyr47, α3: Val69-Leu79, α4: Asn102-Glu113, α5: Pro133-Leu148) on two sides. The FMN-binding site is formed by three loops, namely the P-loop (residues Gly9-Asn14), the 50s-loop (residues Ser57-Ile68) and the 90 s loop (residues Asp94-Cys101). In the apo-form, the three FMN-binding loops are highly mobile and adopt a flexible conformation, as indicated by the missing of many backbone amide signals and the lack of NOE contacts. In the holo-form, the P-loop is responsible for binding the phosphate group, whereas the 50s- and 90s-loops together bind the aromatic flavin ring of the FMN molecule.

Structural comparison of apo- and holo-YqcA shows an essentially identical structure core (Figure 1E), with a root mean square deviation (r.m.s.d.) value of 1.38 Å for backbone atoms of all residues. The most significant conformational differences between these two forms are observed at the cofactor binding loops, especially the P-loop and the 50 s loop. These loops are well defined in the holo-form, whereas they show considerable flexibility in the apo-form (Figure 1A. Notably, among the 23 missing residues in the ^15^N-edited HSQC spectrum of the apo-form, 10 are located in these two loops, suggesting intermediate conformational exchanges on the NMR timescale. In addition, we also observed the extension of secondary structural elements in the holo-form. In particular, helix α1 extends three residues at the N-terminal towards the FMN-binding pocket upon FMN binding.

### NMR characterizations of *E. coli* FldA

Similar to YqcA, the FldA protein directly purified from *E. coli* also showed two sets of cross peaks in the 2D HSQC spectrum, indicating the coexistence of multiple conformations. By using the trichloroacetic acid precipitation and refolding procedures, we were able to obtain FldA samples in the pure apo- and holo-forms, respectively. FMN titration experiments were performed with FldA, and we similarly observed peak disappearance of the apo-form, and the appearance of a new set of peaks corresponding to the holo-form. Notably, the 2D HSQC spectrum of apo-FldA showed significant peak broadening and about 30% of the backbone signals were missing, suggesting extensive conformational exchanges on the intermediate timescales.

We subsequently assigned the chemical shifts of both apo- and holo-FldA. Near complete assignments for backbone and side chain atoms were obtained for holo-FldA (Figure S2), which are consistent to the data previously reported [18]. However, due to substantial loss of peaks of the apo-form, only backbone chemical shifts were assigned for the observable peaks in the HSQC spectrum (Figure S3). The coordinates of holo-FldA are deposited in the PDB under the accession number 2MOK, and the structural statistics are summarized in Table S2.

**Figure 2:**
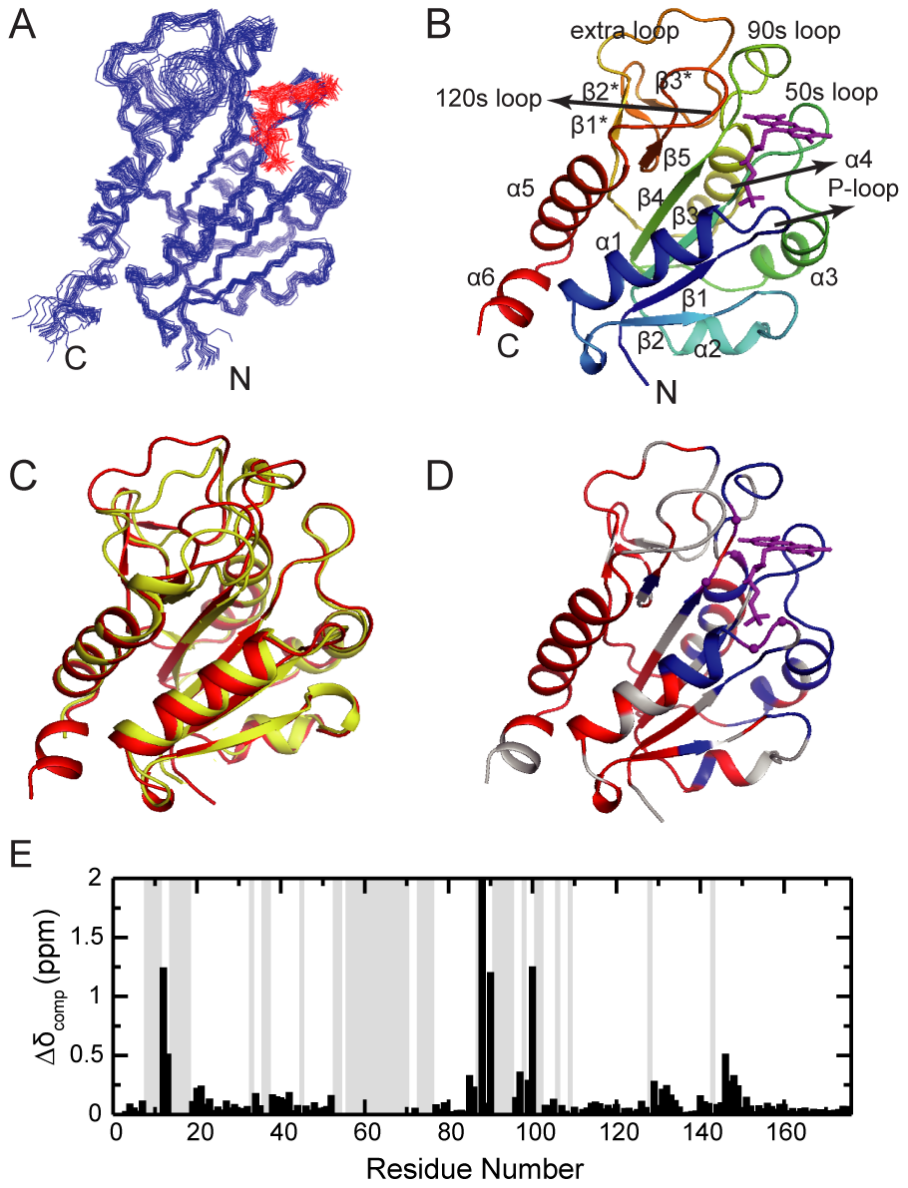
Structural characterizations of the apo- and holo-forms of *E. coli* FldA. (A) Superimposition of the 20 representative solution structures of holo-FldA. The FMN molecule is shown in red. (B) Ribbon diagram representation of holo-FldA, with secondary structural elements and loops labeled. (C) An overlay of the ribbon diagram of the solution structure (red) and crystal structure (yellow, PDB code 1AHN). The FMN molecule is not shown. (D–E) Chemical shift differences of the apo- and holo-FldA (E) and mapping onto the holo-FldA structure (D). The composite chemical shift changes were calculated using the empirical equation, 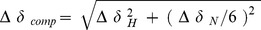, where Δδ_H_ and Δδ_N_ are the chemical shift changes of ^1^H and ^15^N, respectively. The grey-colored background columns in (E) and the blue-colored segments in (D) represent the missing residues in apo-FldA. The residues showing little changes in chemical shifts (Δδ_comp_<0.1 ppm) are colored red in (D). The residues showing large chemical shift changes (Δδ_comp_>0.5 ppm) are show as purple balls in (D).

The solution structures of holo-FldA were solved and shown in Figure 2A–C. The structure is generally similar to the previously reported crystal structure of holo-FldA [17], showing a backbone r.m.s.d value of 1.74 Å. It consists a typical long-chain flavodoxin fold, comprising a central five-strand parallel β-sheet (β1: Thr4-Phe8, β2: Ala-31-Asp35, β3: Ile49-Gly53, β4: Leu82-Gly87, β5: Thr115-Val117 and Leu142-Ala143) flanked by five α-helices (α1: Asn14-Leu26, α2: Lys41-Ala46, α3: Cys64-Leu73, α4: Ala101-Ile109, α5: Thr153-Glu167) on two sides. The β5 was split in the middle by the 20 amino acid insertion (His119-Gly141) unique to the long-chain flavodoxin subfamily. This extra sequence forms an additional small three-strand β-sheet (β1*: Trp120-Gly122, β2*: Leu133-Asp135, β3*: His138-Phe139). Moreover, residues Asp171-Leu174 at the C-terminal tail form a small four-residue helix α6. Similarly, the FMN-binding loops consist the P-loop (residues Gly9-Gly13), 50 s loop (residues Ile54-Gln63) and the 90 s loop (residues Cys88-Asp100). Residues Thr122-Gly132 in the insertion sequence form a relatively long loop (termed the ‘extra loop’ hereafter), which is packed close to the outside of the 90 s loop but does not directly involve in FMN binding.

Due to the severe signal broadening of the NMR spectra, we were unable to calculate the solution structure of apo-FldA. Instead, we tried to characterize it by mapping the chemical shift differences between the apo- and holo-forms onto the holo-structure. As shown in Figure 2D–E, signals that were missing in the apo-form (shown in grey) are clustered around the FMN-binding pocket. In addition to the FMN-binding loops that directly contact the cofactor, neighboring regions including the C-termini of the five β-strands, as well as the N-termini of helices α1-α4, also show significant signal loss. Among the backbone amides peaks that are present in both forms, notable chemical shift differences between the two states are observed for residues Thr12, Gly13, Cys88, Asp90, Asp100 and Glu146, which are all located in the FMN-binding loops. Conversely, the chemical shifts of the remaining residues show rather similar chemical shifts in the two forms, indicating similar chemical/conformational environment in the two states. These residues locate in regions far away from the FMN-binding pocket, including the N-termini of the central β-strands, helices α5-α6, and the small β-sheet formed by the 20 amino acid insertion. These results together suggest that the structure core is partially preserved in apo-FldA, while the regions around the cofactor-binding pocket undergo extensive conformational exchanges on the intermediate timescales.

### Backbone dynamics of YqcA

Since protein functions strongly rely on not only the static structure but also the motional flexibility, we used solution NMR method to further investigate the backbone dynamics of YqcA in both its apo- and holo-forms. The ^15^N backbone relaxation parameters, including the longitudinal relaxation rates *R_1_*, the transverse relaxation rates *R_2_*, and the heteronuclear Overhauser effect {^1^H}-^15^N NOE values were measured for both apo- and holo-YqcA, and analyzed using the Model-free formalism [40], [41]. The relaxation data have been deposited to the BMRB under the accession numbers 25013 and 25014.

**Figure 3:**
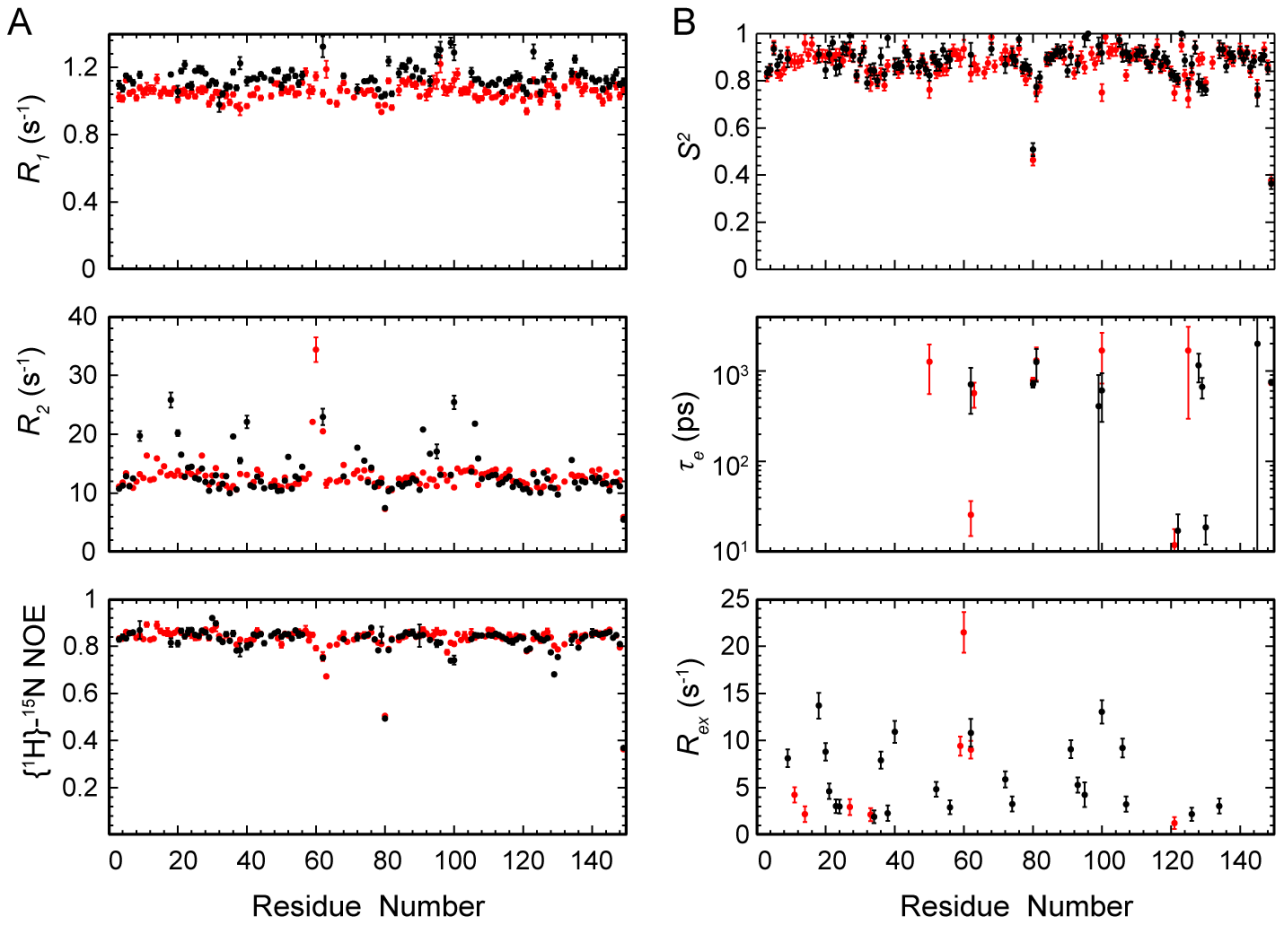
Backbone relaxation data and internal dynamic parameters of *E. coli* YqcA. (A) Longitudinal relaxation rates (*R*_1_), transverse relaxation rates (*R*_2_), and heteronuclear {^1^H}-^15^N NOE values of the apo- (black) and holo-form (red) of *E. coli* YqcA *versus* the amino acid sequence. The data were recorded on a Bruker Avance 800-MHz spectrometer at 25°C. (B) The backbone dynamic parameters *S^2^*, *τ_e_*, and *R_ex_* of the apo- (black) and holo-form (red) of *E. coli* YqcA *versus* the amino acid sequence.

During the data analysis, 107 out of 149 residues were used for the apo-form, whereas 136 were used for the holo-form (Figure 3A). The unanalyzed residues include the proline residues, the ones unassigned, overlapped or too weak to be accurately analyzed. The diffusion tensors for both forms are best represented by the axially symmetric model. For apo-YqcA, the rotational correlation time is *τ_m_* = 7.36±0.02 ns, and the diffusion anisotropy is *D_∥_/D_⊥_* = 1.15±0.02. For holo-YqcA, the rotational correlation time is *τ_m_* = 7.89±0.02 ns, and the diffusion anisotropy is *D_∥_/D_⊥_* = 1.19±0.02. The results indicate that both forms of YqcA exist as monomers in solution.

In the subsequent model-free analyses, five models with increasing complexity (M1, *S^2^*; M2, *S^2^*, *τ_e_*; M3, S^2^, *R_ex_*; M4, *S^2^*, *τ_e_*, *R_ex_*; M5, *S_f_^2^*, *S^2^*, *τ_e_*) were used iteratively to reproduce the experimental data until confidence reached within 95% [40], [41]. The extracted internal mobility parameters, including the generalized order parameter *S^2^* describing the amplitude of internal motions, the effective correlation time *τ_e_* describing the rate of internal motions on the picosecond to nanosecond (ps-ns) timescales, and the *R_ex_* describing the conformational exchanges on the micro- to millisecond (µs-ms) timescales are shown in Figure 3B.

For apo-YqcA, a total of 75 residues were assigned to model M1, with an average *S^2^* = 0.89±0.05. Two residues (Met122 and Asn130) were assigned to model M2, with an average *S^2^* = 0.78±0.02 and internal motions on the ps-ns timescales. Twenty-one residues (Gly9, Val18, Glu20, Glu21, Glu23, Ala24, Thr34, Phe36, Asp38, Glu40, Val52, Thr56, Phe72, Gly74, Ala91, Gly93, Ser95, Gln106, Phe107, Asp126 and Glu134) were assigned to model M3, with an average *S^2^* = 0.90±0.04 and conformational exchanges (*R_ex_*) on the µs-ms timescales. Two residues (Gly62 and Phe100) were assigned to model M4, whereas five residues (Gly80, Phe81, Ser128, Glu129 and Ser149) were assigned to model M5.

For holo-YqcA, a total of 121 residues were assigned to model M1, with an average *S^2^* = 0.88±0.05. Only one residue (Asp63) was assigned to model M2. Six residues (Met11, Asn14, Thr27, Ala33, Thr59 and Gly60) were assigned to model M3, with an average *S^2^* = 0.90±0.06 and conformational exchanges (*R_ex_*) on the µs-ms timescales. Two residues (Gly62 and Glu121) were assigned to model M4, whereas five residues (Gly80, Phe81, Phe100, Ile125 and Ser149) were assigned to model M5.

**Figure 4:**
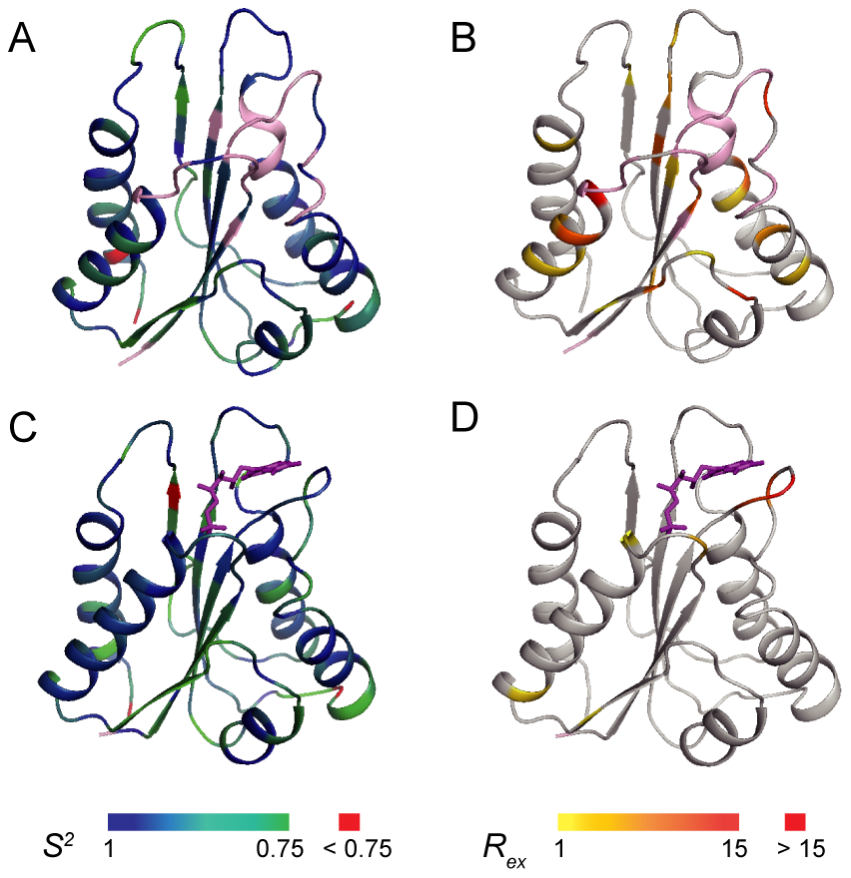
Mapping of the internal dynamics of *E. coli* YqcA onto the structures. (A, C) Ribbon diagrams of the apo- (A) and holo-YqcA (C) representing the generalized order parameter *S^2^* values. Colors ranging from green to blue correspond to *S^2^* values from 0.75 to 1, and red corresponds to *S^2^*<0.75. Missing residues in apo-YqcA are shown in pink. (B, D) Ribbon diagrams of the apo- (B) and holo-YqcA (D) representing the *R_ex_* values. Colors ranging from yellow to red correspond to *R_ex_* values from 1 s^−1^ to 15 s^−1^. Residues with *R_ex_*>15 s^−1^ are also shown in red. Missing residues in apo-YqcA are shown in pink.

The extracted dynamic parameters are mapped onto the YqcA structures in both forms as shown in Figure 4. Overall, the core structures of apo- and holo-forms of YqcA display a relatively high rigidity, as the residues in the secondary structural elements generally show high *S^2^* values. However, holo-YqcA contains more residues that could be described by model M1, and the *S^2^* values are generally higher compared to that of the apo-form YqcA. In addition, significant conformational exchanges on the µs-ms timescales were observed around the FMN-binding pocket in apo-YqcA (Figure 4B). Moreover, the missing of backbone amide signals for residues in the FMN-binding site in apo-YqcA is also an indication of conformational exchanges on the intermediate timescales. After FMN binding, backbone amide signals of many residues become observable, and the conformational exchanges around the binding site are largely decreased (Figure 4D). On the other hand, the holo-YqcA is not entirely rigid. In particular, conformational exchanges on µs-ms timescales are observed for residues Thr59, Gly60 and Gly62 in the FMN-binding 50 s loop.

### Backbone dynamics of FldA

The ^15^N backbone relaxation parameters were similarly measured for both apo- and holo-FldA. Considering the fact that the structure of apo-FldA is unavailable, we employed the reduced spectral density mapping method to analyze the dynamic properties of apo-FldA. For holo-FldA, since both crystal and solution structures are available, we analyzed the relaxation data using the reduced spectral density mapping method for comparison with the apo-form, while used the model-free formalism to obtain further dynamic information of the holo-FldA and for comparison with the short-chain flavodoxins. The relaxation data of holo-FldA have been deposited to the BMRB under the accession number 25015.

**Figure 5:**
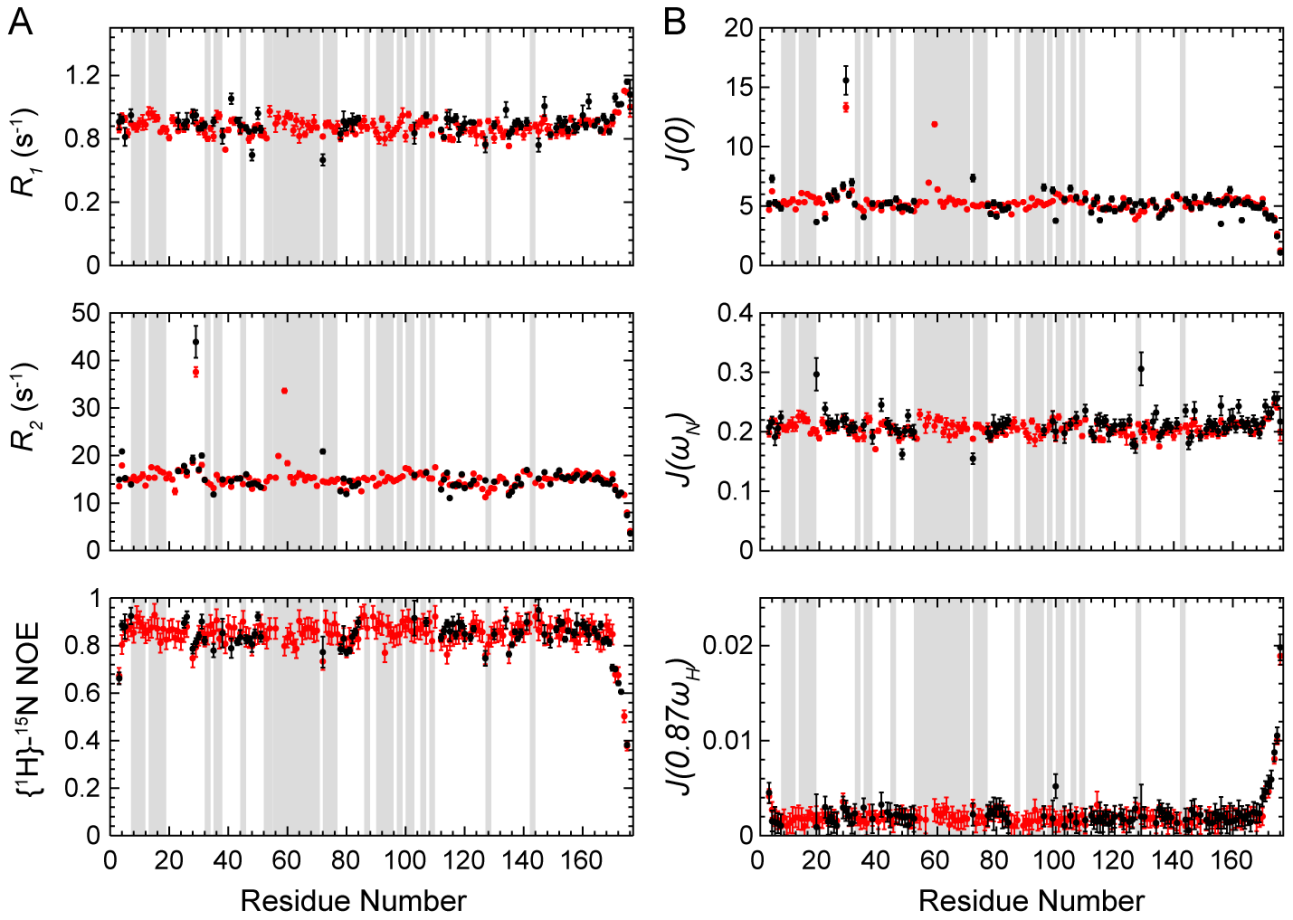
Backbone relaxation data and reduced spectral density functions of *E. coli* FldA. (A) Longitudinal relaxation rates (*R*_1_), transverse relaxation rates (*R*_2_), and heteronuclear {^1^H}-^15^N NOE values of the apo- (black) and holo-FldA (red) *versus* the amino acid sequence. The data were recorded on a Bruker Avance 800-MHz spectrometer at 25°C. (B) The extracted spectral density functions *J(0)*, *J(0.87ω_H_)* and *J(ω_N_)* of the apo- (black) and holo-FldA (red) *versus* the amino acid sequence. The grey-colored background columns in both panels represent the missing residues in apo-FldA.

Figure 5 shows the relaxation data and the extracted spectral density functions of FldA in both forms. The *J(0.87ω_H_)*) and *J(ω_N_)* values reflect the internal motions on the ps-ns timescales, while the *J(0)* value is mainly affected by the transverse relaxation rate *R_2_* and can reflect conformational exchanges on the µs-ms timescales or motional anisotropy. For residues that locate far away from the FMN-binding site, the spectral density functions show overall similarity in both apo- and holo-FldA. For example, the C-terminal helix α6 shows high flexibility on the ps-ns timescales in both apo- and holo-forms, whereas the residues in the secondary structural elements are generally rigid. The fact that most residues in the FMN-binding loops are missing in the apo-form is an indication of conformational exchanges. These slow timescale motions are largely suppressed upon FMN binding, since the signals mostly show up and display spectral density function generally similar to residues in the secondary structural elements. In addition, several residues in the 90 s loop and the extra loop (residues Asn100 and Ala129) undergo fast timescale motions in the apo-form, which are also suppressed in the holo-form. However, two residues (Trp57 and Tyr59) in the 50 s loop show significantly elevated *J(0)* values in the holo-form, indicating motions on the µs-ms timescales, which could also be suggested from the elevated *R_2_/R_1_* and R_2_*R_1_ values (Figure S4).

**Figure 6:**
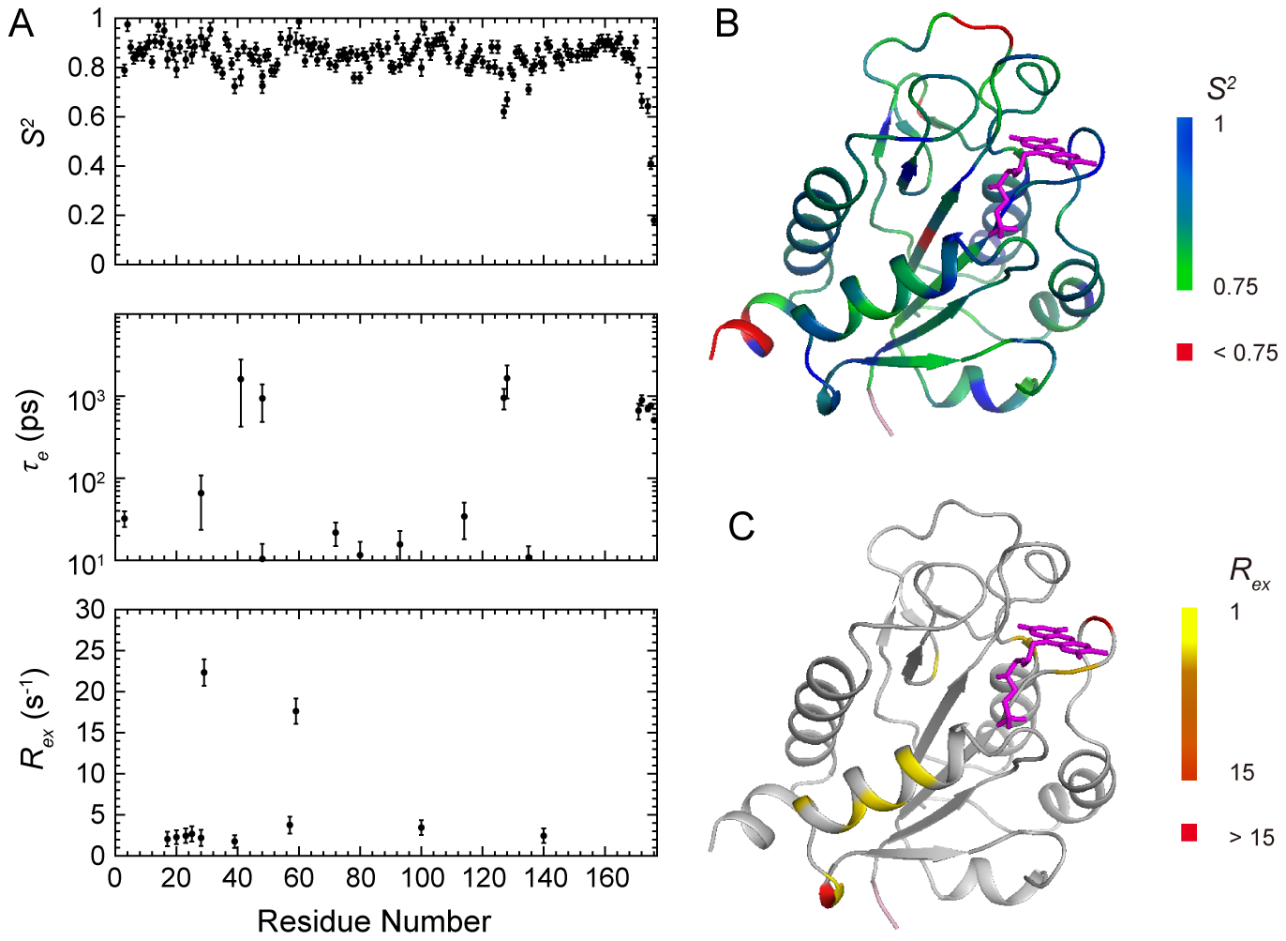
Internal dynamic parameters of holo-FldA. (A) The backbone dynamic parameters *S^2^*, *τ_e_*, and *R_ex_* of holo-FldA *versus* the amino acid sequence. (B) Ribbon diagrams of holo-FldA representing the generalized order parameter *S^2^*. Colors ranging from green to blue correspond to *S^2^* values from 0.75 to 1, and red corresponds to *S^2^*<0.75. (C) Ribbon diagrams of holo-FldA representing the *R_ex_* values. Colors ranging from yellow to red correspond to *R_ex_* values from 1 s^−1^ to 15 s^−1^. Residues with *R_ex_*>15 s^−1^ are also shown in red.

The model-free analysis results of holo-FldA are shown in Figure 6. Briefly, the diffusion tensor of holo-FldA is best represented by the axially symmetric model. The rotational correlation time is *τ_m_* = 9.73±0.03 ns, and the diffusion anisotropy is *D_∥_/D_⊥_* = 1.05±0.02, indicating the monomeric conformation. A total of 135 residues were assigned to model M1, with an average *S^2^* = 0.86±0.02. Seven residues (Ile3, Asp48, Thr72, Gly80, Asp93, Ala114 and Asp135) were assigned to model M2, with an average *S^2^* = 0.79±0.02 and internal motions on the ps-ns timescales. Ten residues (Asn17, Lys20, Gln23, Gln25, Asp29, Ser39, Trp57, Tyr59, Asp100 and Val140) were assigned to model M3, with an average *S^2^* = 0.83±0.03 and conformational exchanges (*R_ex_*) on the µs-ms timescales. Only one residue Lys28 was assigned to model M4, whereas eight residues (Lys41, Asp48, Phe127, Glu128, Asp171, Glu172 and Leu174-Ala176) were assigned to model M5 with an average *S^2^* = 0.60±0.03.

The holo-FldA shows overall structural rigidity, as reflected by the high *S^2^* values and the large number of residues that can be described by model M1. However, the structure is not completely rigid, and conformational exchanges are observed for some residues in helix α1 and the 50 s loop. In particular, residues Asn17, Lys20, Gln23 and Gln25 in helix α1, and residues Trp57 and Tyr59 in the 50 s loop are assigned to M3. The observation that the 50 s loop still undergoes conformational exchanges in the presence of bound cofactor is similar to that of YqcA.

## Discussion

**Figure 7:**
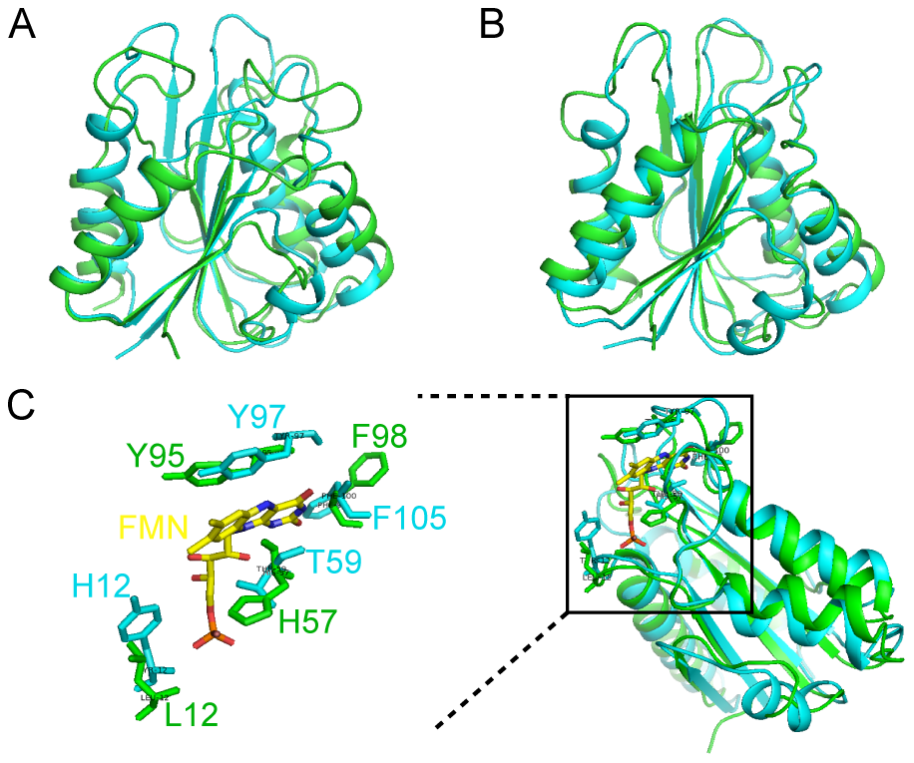
Structural comparisons. (A) A structural overlay of ribbon diagrams of apo-YqcA (cyan) and apo-MioC (green). (B) A structural overlay of ribbon diagrams of the holo-YqcA (cyan) and holo-MioC (green). (C) Local conformational differences around the FMN-binding pocket between YqcA and MioC.

Our current studies on the structures and binding activities reveal that *E. coli* YqcA adopts a typical flavodoxin fold and binds the FMN molecule with a high affinity. These results, in combination with the bioinformatics analysis, strongly support that YqcA is a member of the short-chain flavodoxin subfamily. As a part of our systematic investigations on *E. coli* flavodoxins, we have previously reported the solution structures and backbone dynamics of another *E. coli* short-chain flavodoxin MioC by NMR spectroscopy [17]. A comparison of the structures between YqcA and MioC shown in Figure 7A–B. The two proteins share a similar fold, and the secondary structural segments can all be well imposed upon each other. The r.m.s.d values of the aligned Cα atoms are 3.2 Å and 2.1 Å for the apo- and holo-forms, respectively. Local conformational differences are observed at the FMN binding site. For example, the 50 s loop of YqcA contains fewer aromatic residues than MioC, with a His57 in MioC substituted by a Thr59 in the equivalent position (Figure 7C). In addition, the P-loop of YqcA contains an aromatic residue Tyr12 which is generally absent in the sequences of other flavodoxins. These could affect the redox potentials and interaction specificities of the two proteins and thus the functional differentiation *in vivo*. However, the function of the YqcA protein is yet unknown and remains to be further investigated.

The FMN-binding loops in both YqcA and MioC proteins show higher conformational heterogeneity in the apo-form, while this conformational dynamics becomes largely suppressed upon cofactor binding. Moreover, extensions of secondary structural elements upon FMN binding are also observed in both cases. Similar phenomenon was also observed for the FldA protein of the long-chain subfamily. However, the apo-form of FldA exhibits more severe conformational flexibility, with about one third of the backbone signals missing. In the past decades, extensive efforts have been made to characterize the structures of apoflavodoxins using both X-ray crystallography and NMR spectroscopy. Whereas the X-ray structures of apoflavodoxins show a compact fold with the 50 s loop adopting a closed conformation [42]–[43], NMR studies of *Azotobacter vinelandii* and *Anabaena* apoflavodoxoins [44]–[45], simulation data of *H. pylori* apoflavodoxin [43] and an alternative conformation observed in the X-ray structure of a mutant *Anabaena* apoflavodoxin [46] highlighted the flexibility of the cofactor binding loops, particularly the 50 s loop which binds the isoalloxazine ring of the FMN molecule. These previously published results together with our studies suggest that significant conformational exchanges of the FMN-binding loops in the apo-form and the stabilization after FMN binding are common features for the flavodoxin family. The scenario that the cofactor binding loops sample multiple conformations in the apo-form better explains the kinetic observations that binding to the isoalloxazine ring instead of the phosphate group initiates the apoflavodoxin-FMN complex formation [46]–[47]. Binding of the FMN molecule to the relatively flexible loops completes the final step of the flavodoxin folding event, and stabilizes the whole protein to an energetically more favorable state as suggested by Bollen et al [48].

On the other hand, although YqcA, FldA and MioC are all significantly stabilized after FMN binding, backbone dynamic investigations indicate that certain residues in the 50 s loops still exhibit slow timescale conformational exchanges in the holo-form in all cases, such as residues Thr59, Gly60, and Gly62 for holo-YqcA, residues Trp57 and Tyr59 for holo-FldA, and residues Ala59-Ile62 for holo-MioC. Since flavodoxins are a model system for studies of protein folding and cofactor binding, they have been subjected to various biochemical and structural characterizations, including several early dynamics investigations by solution NMR technique [49]–[51]. The backbone dynamics study of the *Desulfovibrio vulgaris* flavodoxin by Hrovat et al also revealed higher than average *R_2_/R_1_* values at the 50 s loop region, which is also an indication of conformational exchanges on the slow timescales [51]. Intriguingly, mutagenesis and crystallographic studies showed that the flexibility of the 50 s loop plays an important role in the redox redactions of flavodoxins. In particular, in the studies of *Clostridium beijerinckii* flavodoxin, a backbone conformational flip involving the dipeptide Gly57-Asp58 was observed in the transition of the FMN molecule from the fully-oxidized state to the semi-quinone state, accompanied by the formation of a new hydrogen bond [52]–[54]. Similar phenomenon was also observed for *D. Vulgaris* flavodoxin [55]. Further experimental data suggested that lacking of the flexible glycine residue in the 50 s loop would severely affect the redox potential, thus supporting the importance of the conformational flexibility of this loop in the redox reactions [56]. Our current backbone dynamics studies of holo-YqcA and holo-FldA, together with previous reports on MioC [17] and *D. Vulgaris* flavodoxin [51], demonstrate that the µs-ms timescale conformational exchanges in the 50 s loop are commonly observed in the holo-form of both long-chain and short-chain flavodoxins. Since the holo-proteins used in these studies all contain oxidized FMN molecule, the observed conformational dynamics suggest that the 50 s loop samples multiple conformational spaces in this single redox state, and the local conformational flexibility facilitates the fine-tuning and adaption of the protein structure to other redox states during electron transfer. Notably, the backbone dynamics of the *Anacystis nidulans* holoflavodoxin revealed an unusual lack of internal flexibility throughout the protein sequence, including the 50 s loop [49]. This flavodoxin exhibits a redox potential for the oxidized/semiquinone transition close to that of free flavin, and a much more negative redox potential for the semiquinone/hydroquinone transition [49], whereas most flavodoxins significantly alter both redox potentials of the oxidized/semiquinone and semiquinone/hydroquinone transitions [11], [57]. This distinct observation strongly suggests a possible connection between protein dynamics and the modulation of FMN redox potentials, as has been previously suggested [56]. However, further investigations on flavodoxin conformational dynamics are expected to clearly establish its role in redox potential modulations.

In summary, our current structural and dynamics studies on YqcA and FldA reveal significant conformational exchanges around the cofactor-binding site in their apo-forms. In contrast, upon FMN binding, the holo-forms are largely stabilized, while the 50 s loop still displays conformational flexibility. These results, together with previously published studies on other flavodoxins, suggest that these are common features among both long-chain and short-chain flavodoxins, and are relevant to their FMN binding activities and redox reaction processes. The observations in our current study and in literature suggest a conformational selection mechanism for both FMN binding and redox transfer reactions. We speculate that the conformations sampled by FMN-binding loops in the apoflavodoxins may include those more favorable for FMN interaction, thus facilitating the binding process, whereas the conformations sampled by the 50 s loop in the oxidized holoflavodoxins may include those favorable for interactions with FMN in the semiquinone or hydroquinone states, thus facilitating the electron transfer steps. Further investigations are expected to clarify this hypothesis.

## Supporting Information

Figure S1 **2D ^1^H-^15^N HSQC spectrum of directly purified YqcA showing two sets of peaks.** Representative residues with clear distinction of the two sets of peaks are labeled in red for the holo-form and blue in the apo-form.
(TIF)

Figure S2 **2D ^1^H-^15^N HSQC spectrum of holo-FldA.** The assignments are annotated with the one-letter amino acid code and the sequence number. The side-chain NH_2_ peaks of Asn and Gln are connected by horizontal lines.
(TIF)

Figure S3 **2D ^1^H-^15^N HSQC spectrum of apo-FldA.** The assignments are annotated with the one-letter amino acid code and the sequence number. The side-chain NH_2_ peaks of Asn and Gln are connected by horizontal lines.
(TIF)

Figure S4 **Backbone relaxation parameters of** ***E. coli*** **FldA.** Backbone ^15^N *R*_1_*R_2_ and *R*_2_/*R*_1_ values of the apo- (black) and holo-FldA (red) *versus* the amino acid sequence. The grey-colored background represents the missing residues in apo-FldA. The data were recorded on a Bruker Avance 800-MHz spectrometer at 25°C.
(TIF)

Table S1 **Structural statistics of** ***E. coli*** **YqcA.**
(PDF)

Table S2 **Structural statistics of** ***E. coli*** **holo-FldA.**
(PDF)
