# Supplementary figures and images for: Conformational Dynamics of Escherichia coli Flavodoxins in Apo- and Holo-States by Solution NMR Spectroscopy
Source: PLoS One. 2014 Aug 5;9(8):e103936. doi: 10.1371/journal.pone.0103936 (PMC4122359; doi:10.1371/journal.pone.0103936)

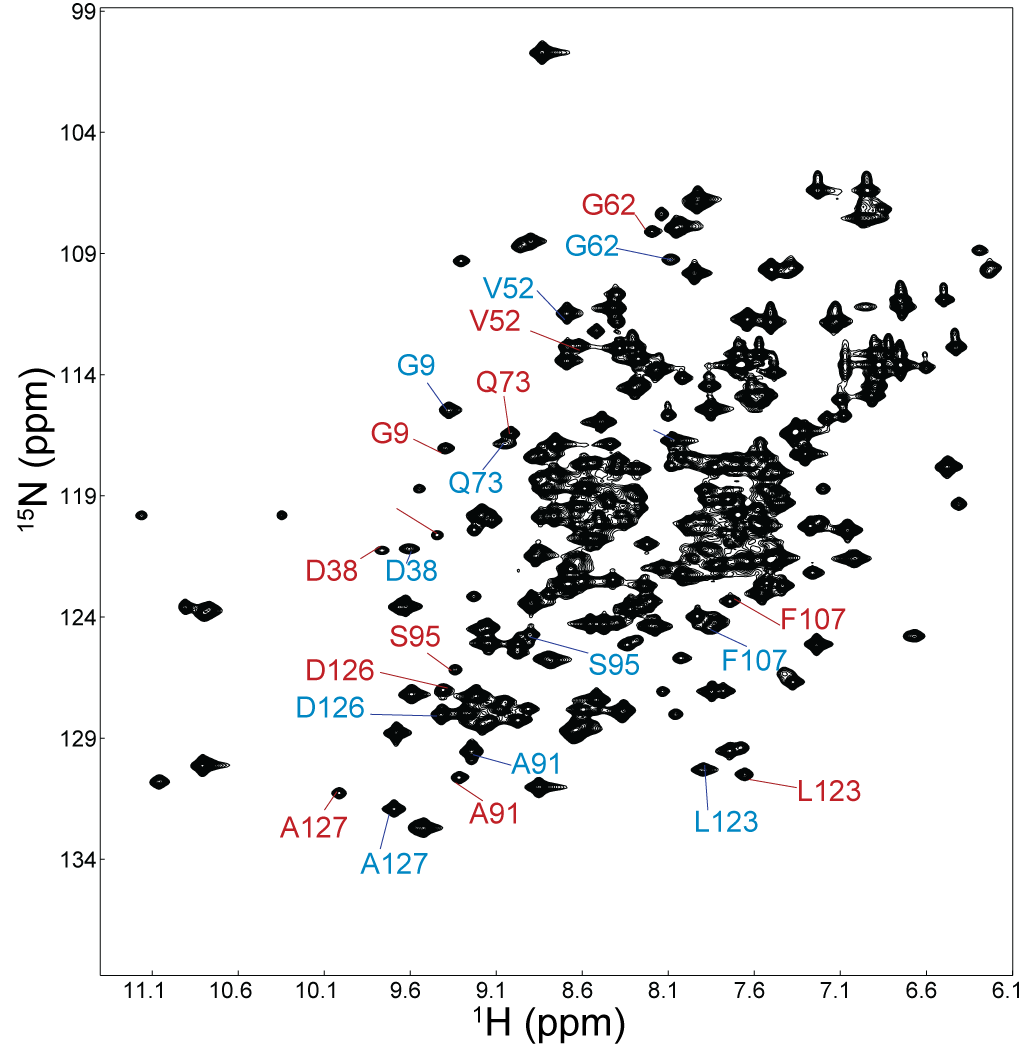

Supplement: Figure S1 — 2D 1H-15N HSQC spectrum of directly purified YqcA showing two sets of peaks. Representative residues with clear distinction of the two sets of peaks are labeled in red for the holo-form and blue in the apo-form. (TIF) [file pone.0103936.s001.tif]

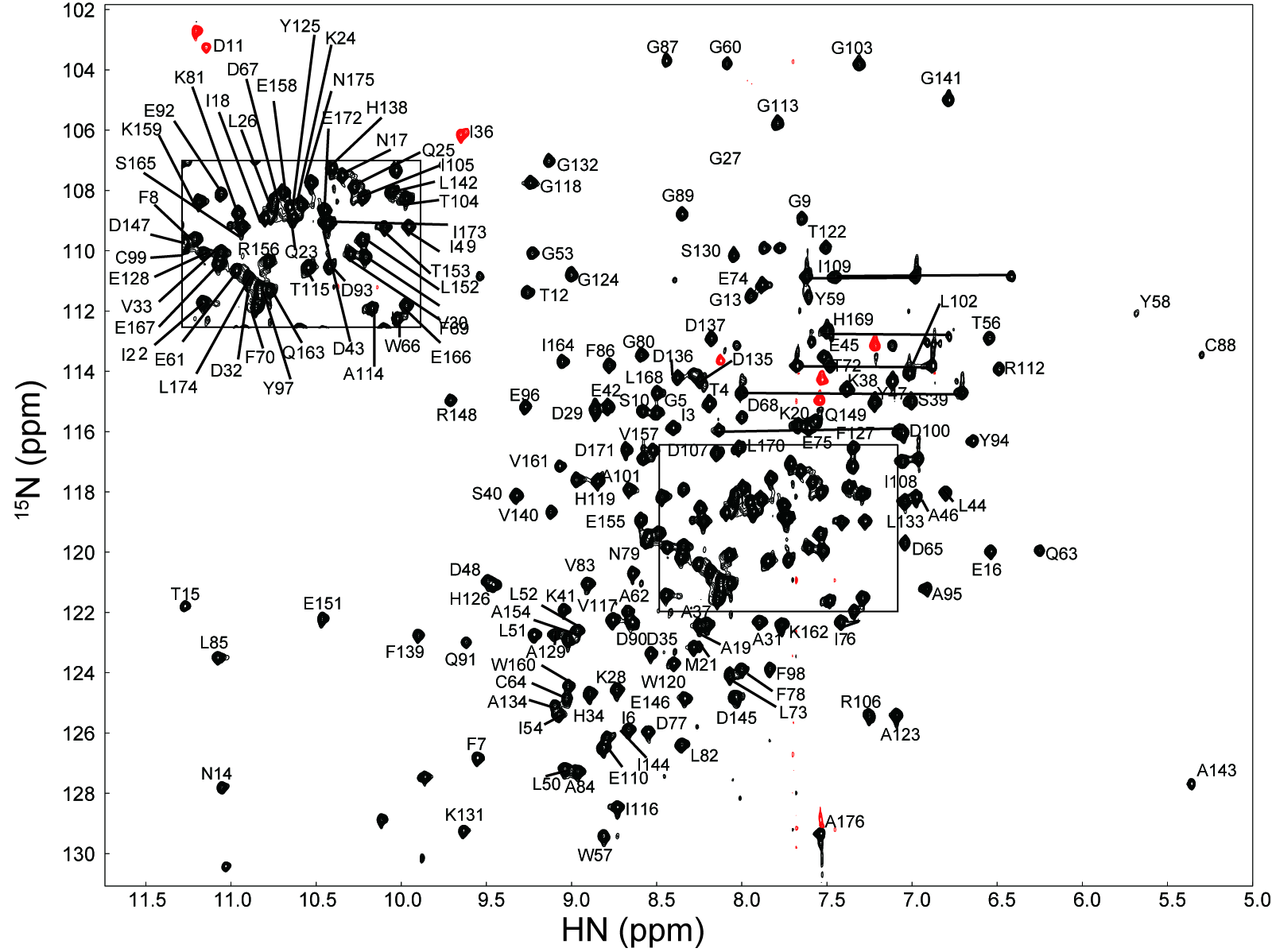

Supplement: Figure S2 — 2D 1H-15N HSQC spectrum of holo-FldA. The assignments are annotated with the one-letter amino acid code and the sequence number. The side-chain NH2 peaks of Asn and Gln are connected by horizontal lines. (TIF) [file pone.0103936.s002.tif]

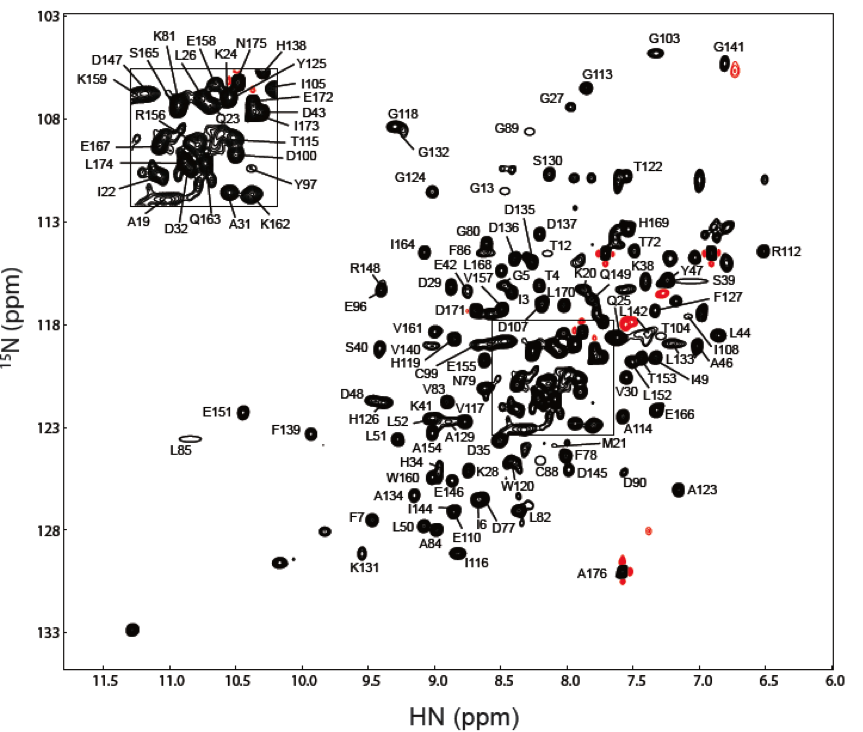

Supplement: Figure S3 — 2D 1H-15N HSQC spectrum of apo-FldA. The assignments are annotated with the one-letter amino acid code and the sequence number. The side-chain NH2 peaks of Asn and Gln are connected by horizontal lines. (TIF) [file pone.0103936.s003.tif]

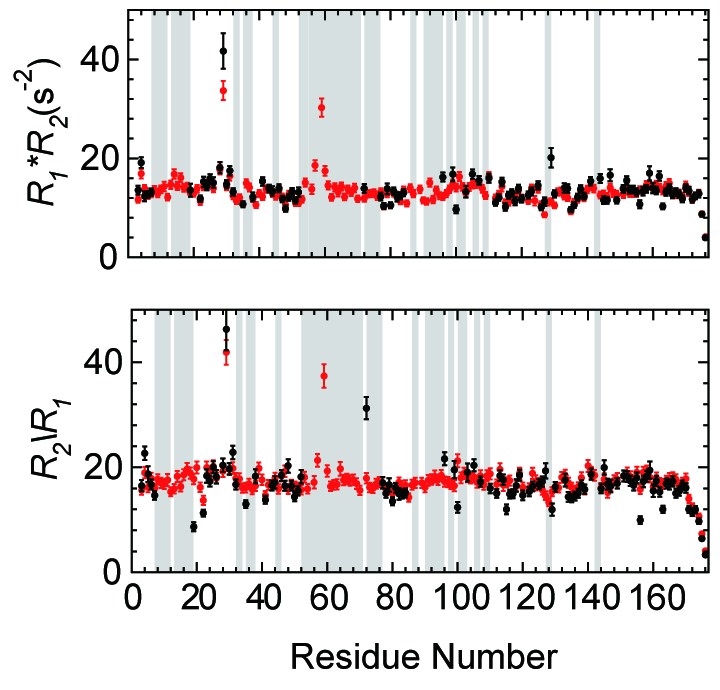

Supplement: Figure S4 — Backbone relaxation parameters of E. coli FldA. Backbone 15N R 1*R 2 and R 2/R 1 values of the apo- (black) and holo-FldA (red) versus the amino acid sequence. The grey-colored background represents the missing residues in apo-FldA. The data were recorded on a Bruker Avance 800-MHz spectrometer at 25°C. (TIF) [file pone.0103936.s004.tif]
