# Supplementary material for: Conformational Dynamics of Escherichia coli Flavodoxins in Apo- and Holo-States by Solution NMR Spectroscopy
Source: PLoS One. 2014 Aug 5;9(8):e103936. doi: 10.1371/journal.pone.0103936 (PMC4122359; doi:10.1371/journal.pone.0103936)
Supplement: Table S1 — Structural statistics of E. coli YqcA. (PDF) [file pone.0103936.s005.pdf]

Table S1. Structural statistics of *E. coli* YqcA

	Apo-YqcA	Holo-YqcA
**Structural restraints**		
Protein intramolecular NOEs		
Total unambiguous NOEs	5987	6027
Intra-residue	2306	2056
Sequential (|i-j| = 1)	1053	1471
Medium-range (1 < |i-j| < 5)	696	948
Long-range	1932	1552
Total ambiguous NOEs	1161	1250
Protein-FMN intermolecular distance restraints	-	22
Dihedral angle restraints ($\phi + \psi$)	124 (62+62)	123 (62+61)
**Restraint violations**		
Distance (> 0.3 Å)	0	0
Dihedral angle (>5°)	0	0
**r.m.s.d. from mean structure (Å)**		
Secondary structure backbone atoms	0.5 ± 0.1	0.3 ± 0.1
Secondary structure heavy atoms	0.8 ± 0.1	0.6 ± 0.1
All backbone atoms	0.7 ± 0.1	0.4 ± 0.1
All heavy atoms	1.1 ± 0.1	0.8 ± 0.1
**Ramachandran statistics (%)**		
Residues in most favored regions	84.2	81.2
Residues in additional allowed regions	13.9	17.6
Residues in generously allowed regions	1.5	1.1
Residues in disallowed regions	0.4	0.1
