# Supplementary material for: Conformational Dynamics of Escherichia coli Flavodoxins in Apo- and Holo-States by Solution NMR Spectroscopy
Source: PLoS One. 2014 Aug 5;9(8):e103936. doi: 10.1371/journal.pone.0103936 (PMC4122359; doi:10.1371/journal.pone.0103936)
Supplement: Table S2 — Structural statistics of E. coli holo-FldA. (PDF) [file pone.0103936.s006.pdf]

Table S2. Structural statistics of *E. coli* holo-FldA

	Holo-FldA
**Structural restraints**	
Protein intramolecular NOEs	
Total unambiguous NOEs	5190
Intra-residue	1992
Sequential (|i-j| = 1)	1237
Medium-range (1 < |i-j| < 5)	760
Long-range	1201
Total ambiguous NOEs	1455
Protein-FMN intermolecular distance restraints	32
Dihedral angle restraints ($\phi + \psi$)	182 (91 + 91)
**Restraint violations**	
Distance (> 0.3 Å)	0
Dihedral angle (>5°)	0
**r.m.s.d. from mean structure (Å)**	
Secondary structure backbone atoms	0.4 ± 0.1
Secondary structure heavy atoms	0.8 ± 0.1
All backbone atoms	0.7 ± 0.1
All heavy atoms	1.1 ± 0.1
**Ramachandran statistics (%)**	
Residues in most favored regions	81.6
Residues in additional allowed regions	16.1
Residues in generously allowed regions	1.8
Residues in disallowed regions	0.5
